# Health and Economic Outcomes of Introducing the New MenB Vaccine (Bexsero) into the Italian Routine Infant Immunisation Programme

**DOI:** 10.1371/journal.pone.0123383

**Published:** 2015-04-13

**Authors:** Marcello Tirani, Michela Meregaglia, Alessia Melegaro

**Affiliations:** 1 Postgraduate School of Public Health, Department of Biomedical Sciences for Health, Università degli Studi di Milano, Milan, Italy; 2 CeRGAS—Centre for Research on Health and Social Care Management, Bocconi University, Milan, Italy; 3 Department of Policy Analysis and Public Management & Dondena Centre for Research on Social Dynamics and Public Policy, Bocconi University, Milan, Italy; Universidad Nacional de La Plata., ARGENTINA

## Abstract

**Introduction:**

In January 2013 a novel type of multicomponent protein-based vaccine against group B meningococcal disease was licensed by the European Medicines Agency. With the widespread use of the meningococcal serogroup C conjugate vaccines, serogroup B remains now the major cause of bacterial meningitis and septicaemia in young children in Europe. The aim of this study is to investigate the health and the economic outcomes of MenB vaccine introduction into the Italian routine mass vaccination programme.

**Methods:**

The present work is structured in two main parts. Firstly, we assess the epidemiological burden of group B meningococcal disease using official hospitalisation and notification data from two of the most populated Italian regions (Lombardia and Piemonte) during a 6-year study period (2007-2012). Secondly, we evaluate the cost-effectiveness of the immunisation programme in Italy from the public health payer perspective under base case parameters assumptions and performing a comprehensive sensitivity analysis to assess the robustness and the uncertainty of our model results.

**Results:**

MenB serotype is responsible for 59% of the 341 cases of Invasive Meningococcal Disease in Lombardia and Piemonte. Incidence rate for MenB infection is estimated to be 0.21/100,000/y resulting at the highest level in children ≤4 years of age. Although the new MenB vaccine can potentially prevent about one third of the disease cases in the Italian population, model results show this strategy is unlikely to be cost-effective (ICER value over €350,000/QALY) with a vaccine that prevents disease only. These results are robust under most of the sensitivity scenarios except when allowing for lower discount rates.

**Discussion:**

The introduction of the novel vaccine into the routine immunisation schedule needs to be carefully evaluated. The new MenB vaccine has the potential to reduce the disease burden at the population level. However, from the Italian Health Service perspective, the immunisation programme is unlikely to be cost-effective at the current incidence levels and vaccine price.

## Introduction

*Neisseria meningitidis* continues to be one of the leading infections to cause long-term morbidity and mortality worldwide [1, 2] despite improvements in critical care and availability of effective antibiotics [3, 4]. The main efforts to reduce its incidence and to control its spread are traditionally and primarily focused on prevention through vaccination [5, 6]. In this sense, with the widespread use of the MenC conjugate vaccines, meningococcal serogroup B has become the major cause of bacterial meningitis and septicaemia in young children in Europe [6–9] as well as the responsible of one third of the cases in North America [8, 10–12].

Whilst for the other meningococcal serogroups (A, C, W135, Y) a vaccine was developed on the basis of serogroup-specific capsular polysaccharides [6, 9, 12], up to now a broadly-strain-coverage effective vaccine against capsular group B was not available [8, 12, 13]. A major obstacle to this was that the MenB polysaccharide capsule shares homologous structure with human foetal neural-cell adhesion molecules [14, 15] resulting in poor immunogenic response and raising concerns about the potential for induction of auto-immunity [1, 5, 6].

Different MenB vaccines have been used in the past years to successfully control regional epidemics (New Zealand, Norway, Cuba), but all were strain-specific [16]. These types of vaccine were developed on the MenB outer membrane vesicles (OMVs) [10, 17, 18]. Unfortunately also such vaccines have shown limited efficacy due to the high variability of the immunodominant meningococcal proteins [6, 19, 20].

Novel type of multicomponent vaccines (Bexsero, Novartis, and Trumenba, Pfizer)—able to offer a broader protection against serogroup B *Neisseria meningitidis* and based on sequencing of the whole meningococcal genome to identify surface antigens of the meningococcal strains [21–23]—are now available on the market [2, 5, 24]. One of these, Bexsero, was licensed for use in people older than 2 months by the European Medicines Agency in January 2013 [25] and very recently (January 2015) also by the U.S. Food and Drug Administration [26].

Policy makers now face the decision about whether or not to introduce this novel kind of meningococcal multicomponent vaccine (4CMenB) in their countries and how to eventually fit it into their current National Immunisation Plans (NIPs). As the introduction of new vaccines have important public health implications, a number of key variables (e.g., vaccine efficacy, overall effectiveness, resources consumption, expected compliance and possible interferences within the immunisation schedule in place) needs to be carefully evaluated. This is even more relevant when considering a relatively low incidence disease such as MenB in Italy [27].

Over the next decade several new expensive vaccines will be commercialised and considered for universal use against infectious diseases of major public health importance. The development of new vaccines in the health market arena challenges the current NIPs since it raises concerns of fruitfully prioritising investments [28–32]. Limited financial resources should be distributed in a fair and effective manner in order to achieve the best possible outcomes under local, rather than global, conditions [33] and considering all direct and indirect consequences of the immunisation programme. In this sense, financial constraints might become an opportunity to reconsider prevention as a way to make good investments in health.

The purpose of this study is to investigate the health and the economic outcomes of a potential introduction of the new 4CMenB vaccine (Bexsero) in Italy in order to help inform policy decisions. In details, the current work aims to: 1) assess the epidemiological burden of MenB disease using official hospitalisation and notification data from two of the most populated Italian regions (Piemonte and Lombardia); 2) evaluate the effectiveness and cost-effectiveness of the programme calculating Quality-Adjusted Life Years (QALYs) gained, Net Costs and Incremental Cost-Effectiveness Ratio (ICER) under base case parameters assumptions. Moreover, considering the major uncertainties surrounding the epidemiological and economic parameters, an extended sensitivity analysis is performed.

The CHEERS (Consolidated Health Economic Evaluation Reporting Standards) checklist for reporting economic evaluation analysis was followed [34].

## Materials and Methods

### Analysis of epidemiological data

Invasive Meningococcal Disease (IMD) is a mandatory notifiable disease in Italy, information pertaining to which are available from two data sources: i) the National Invasive Bacterial Diseases (IBD) Surveillance System [35], started in 2007 and using the same case definition adopted by the European Union (2008/426/EC) [36]; ii) the Hospital Discharge Database [37], which covers all admissions to any public or private Italian healthcare facility. For each IMD case, the National IBD Surveillance System provides information about subject’s demographics, clinical manifestations and bacterial features (e.g., capsular group). The Hospital Discharge Database provides data on patients’ demographics (gender, age, place of birth, residence), admission and discharge data, ward and length of stay, status at discharge (alive, dead or transferred to another hospital), primary diagnosis, up to five secondary diagnoses, and up to six medical procedures and surgical interventions. Diagnoses and interventions are reported as code numbers according to the International Classification of Diseases, 9^th^ Revision, Clinical Modification (ICD-9-CM). Hospital activity for ordinary in-patient and day-hospital cases is based on Diagnosis-Related Groups (DRGs) [38]. Therefore inpatient costs were derived using the DRG codes reported for each patient record. These costs include all hospital-related expenditures (i.e., costs for acute standard care and complications).

Patients discharged from any healthcare facilities in the two selected regions (Lombardia and Piemonte) from January 1, 2007, to December 31, 2012, and reporting at least one IMD-related ICD-9-CM code (036.0, 036.1, 036.2, 036.3, 036.4, 036.8, 036.9) were included in the analysis and linked to laboratory-confirmed IMD cases (as extracted from the IBD Surveillance System). In this way hospitalisation records were supplemented with additional information regarding clinical manifestations of the disease and capsular group.

Age and year-specific regional population figures were obtained from the Italian National Statistical Institute (ISTAT) [39] and used as denominators to calculate incidence rates. Age-specific case fatality rates (CFRs) were calculated dividing the number of IMD-related inpatient deaths (as reported by the regional Hospital Discharge Database) by the age-specific total number of admissions.

Descriptive statistical analyses were carried out using SAS version 9.2 (Cary Software, North Carolina SAS Institute Inc. 2004).

### Cost-effectiveness analysis

#### Model structure

**Fig 1:**
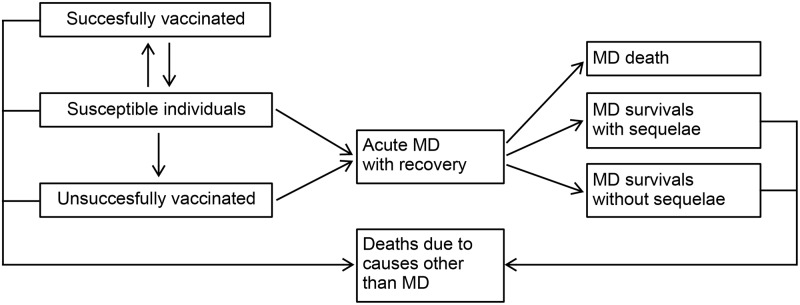
Model structure. Model used to assess the impact of the immunisation programme. Individuals are born in a susceptible unvaccinated compartment. After vaccination individuals move either to the successfully vaccinated state or to the unsuccessfully vaccinated state. The vaccine-induced immunity is considered provisional. Individuals have the chance of developing disease, resulting in either survival without sequelae, survival with sequelae, or death.

A Markov model was constructed to follow two hypothetical (vaccinated and unvaccinated) cohorts of individuals from birth until death (Fig 1). In particular, the 2012 Italian birth cohort (drawn upon ISTAT data [39]) was used to populate the model and stratified into 100 single years of age classes in order to capture the full spectrum of costs and benefits associated to IMD and MenB vaccination over lifetime. Individuals were born into a susceptible unvaccinated state and then exposed to a 3-dose vaccine schedule at 2, 3, 4 months, followed by one catch-up dose between 12 and 23 months. After vaccination individuals could either move from the susceptible state to the successfully vaccinated state or to the unsuccessfully vaccinated state according to vaccine efficacy (VE). A temporary vaccine-induced immunity was considered, thus individuals moved back to the susceptible state after vaccine effect waned; the duration of protection was assumed to be 3 years in the base case analysis. Susceptible and unsuccessfully vaccinated children had the same risk of infection as unvaccinated individuals. The number of IMD cases, by age group and vaccination status, was obtained multiplying the average annual age-specific MenB incidence rate by the age-specific Italian population size in order to obtain the overall number of cases expected at national level and the number of cases avoided as a consequence of the vaccination programme. In line with previous works [5, 40] and epidemiological evidence [41], we supposed individuals could experience the disease once and that the only possible outcomes for each meningitis case were: survival, with or without sequelae, and death, from IMD or other causes.

The number of cases/deaths averted and the number of life years (LYs)/quality-adjusted life years (QALYs) gained from the vaccination were taken as primary measures of outcome of the programme and compared to its net cost (the additional cost of vaccination minus the expected savings from the programme in terms of reduced use of health care resources).

All costs and health benefits were discounted at a 3% annual rate according to the advice of the Italian guidelines [42, 43]. The Markov model was built using Excel 2010 (@Microsoft).

#### Base case model parameters

Details of data and parameters used in the base case model with reference sources are summarised in Tables 1 and 2.

**Table 1:** Base case parameters used in the model.

Parameter	Base case	Distribution	References
Epidemiological parameters			
Disease incidence (per 100,000)	0.21 (variable by age)	Triangular (variable by age and years)	^b^
Case fatality rate (proportion)	0.07 (variable by age)	Beta (variable by age)	^c^
Background mortality rates	Variable by age	Fixed	^d^
Population	Variable by age	Fixed	^d^
Acute treatment parameters			
GP visit cost (€)	16.00	Uniform (12.8; 19.2)	[46,63]
Hospitalisation rate (%)	100	Fixed	Assumed
Cost acute stay, paediatric (≤18 years) (€)^a^	6,800.00	Risk Pearson 5 (4; 20400)	^c^
Cost acute stay, adult (>18 years) (€)^a^	8,250.00	Risk Pearson 5 (6; 40800)	^c^
Long-term parameters			
Survivors with major or minor sequelae (%)	9.50	Beta (1.5; 5.4)	[47]
Cost for those with sequelae (annual, €)	4,147.69	Gamma (3; 920)	[40]
Cost for those without sequelae (first year, €)	489.00	Gamma (15; 33)	[46]
Proportion of different sequelae	See Table 2	See Table 2	[40]
QALY losses for survivors with sequelae	See Table 2	See Table 2	[40]
Vaccine parameters			
Vaccination coverage (%)	80.00	Fixed	^e^
Vaccine efficacy (%)	75.00	Triangular (65; 80; 95)	Assumed
Strain coverage (%)	100	Uniform (84; 100)	[9,57]
Number of doses	4	Fixed	[53]
Duration of protection after 4 doses (years)	3	Triangular (1.5; 3; 4.5)	Assumed
Cost per vaccine dose (€)	67.00	Scenario variation (range 53.6–80.4)	[50,51]
Administration cost per dose (€)	7.00	Gamma (7; 1)	[52]
Rate of mild adverse events (per 10,000 doses)	6.80	Gamma (5.9; 249.5)	[85]
Rate of anaphylactoid events (per 10,000 doses)	0.01	Normal (719,790;112,140)	[85]
Cost for those with mild adverse events (€)	3.40	Gamma (2.8; 1.21)	[40]
Cost for those with anaphylactoid events (€)	1280.75	Gamma (11.85; 98.73)	[86]
Discount rates			
Discount costs (%)	3.00	Scenario variation (range 0–3)	[42,43]
Discount benefits (%)	3.00	Scenario variation (range 0–3)	[42,43]

^a^ The average length of hospital stay was 11.40 days for subjects ≤18 years (paediatric) and 21.64 days for those >18 years (adult).

^b^ Disease incidences by age-class concerning Lombardia and Piemonte regions for the period 2007–2012 were calculated using the numbers of cases collected from the Italian Invasive Bacterial Diseases Surveillance System, the Italian Hospital Discharge Database and the age-specific population of the same period obtained from the Italian National Statistical Institute.

^c^ Data related to MenB case fatality rates and hospital costs were estimated from the Italian Hospital Discharge Dataset.

^d^ Population figures by single year of age and national mortality rates at January 1, 2012, were obtained from the Italian National Statistical Institute.

^e^ Routine immunisation vaccine coverage was assumed similar to the actual one against meningococcal serogroup C in Lombardia and Piemonte regions.

**Table 2:** Probabilities and QALY losses for each single sequela used in the model.

Parameter	Base case	Distribution	References
Proportion of sequelae			
Skin necrosis (%)	1.50	Uniform (1.20; 1.80)	[87]
Amputation with substantial disability (%)	1.00	Uniform (0.80; 1.20)	[88]
Hearing loss with cochlear implantation (%)	2.00	Uniform (1.60; 2.40)	[88]
Moderately severe bilateral hearing loss (%)	5.00	Uniform (4.00; 6.00)	[88]
Any unilateral or bilateral hearing loss (%)	6.00	Uniform (4.80; 7.20)	[88]
Severe neurological disability (%)	2.10	Uniform (1.68; 2.52)	[89]
Mental retard (cognitive problem) (%)	23.70	Uniform (18.96; 28.44)	[87]
Arthritis (%)	2.90	Uniform (2.32; 3.48)	[87]
Epilepsy or seizure (%)	2.00	Uniform (1.60; 2.40)	[88]
Depression (%)	5.70	Uniform (4.56; 6.84)	[87]
Anxiety (%)	7.10	Uniform (5.68; 8.52)	[87]
Blindness (%)	0.40	Uniform (0.32; 0.48)	[88]
Motor deficit (%)	1.90	Uniform (1.52; 2.28)	[2]
Severe speech communication problems (%)	3.80	Uniform (3.04; 4.56)	[88]
Migraine (%)	10.00	Uniform (8.00; 12.00)	[87]
Renal failure (%)	1.90	Uniform (1.52; 2.28)	[87]
QALY losses for survivors with sequelae			
Skin necrosis	0.10	Uniform (0.08; 0.12)	[90]
Amputation with substantial disability	0.39	Uniform (0.31; 0.47)	[89]
Hearing loss with cochlear implantation	0.19	Uniform (0.15; 0.23)	[84]
Moderately severe bilateral hearing loss	0.09	Uniform (0.07; 0.11)	[84]
Any unilateral or bilateral hearing loss	0.28	Uniform (0.22; 0.34)	[84]
Severe neurological disability	0.94	Uniform (0.75; 1.13)	[89]
Mental retard (cognitive problem)	0.46	Uniform (0.37; 0.55)	[91]
Arthritis	0.31	Uniform (0.25; 0.37)	[92]
Epilepsy or seizure	0.17	Uniform (0.14; 0.20)	[84]
Depression	0.27	Uniform (0.22; 0.32)	[93]
Anxiety	0.31	Uniform (0.25; 0.37)	[93]
Blindness	0.74	Uniform (0.59; 0.89)	[94]
Motor deficit	0.17	Uniform (0.14; 0.20)	[95]
Severe speech communication problems	0.61	Uniform (0.49; 0.73)	[96]
Migraine	0.19	Uniform (0.15; 0.23)	[97]
Renal failure	0.18	Uniform (0.14; 0.22)	[98]

As reported in the literature [5, 44, 45] and as a result of our empirical comparison of the two IMD datasets (showing that all recorded patients were admitted to the hospital), we assumed a 100% MenB hospitalisation rate. On top of this, and following Italian evidence from Lucioni and colleagues [46], a GP consultation occurring either before or after admission to the hospital was included in the current analysis. Hospitalised patients were assumed to be at risk of death according to their age-specific CFR.

Model transition probabilities and QALY losses associated to long-term sequelae were assigned to the main IMD complications on the basis of an extensive structured literature review performed in a 2013 Italian Health Technology Assessment (HTA) evaluation paper by di Pietro at al [40]. Since multiple complications are rare [47–49], we assumed that their probabilities were mutually independent.

The average cost of a meningococcal disease case is a composite measure of the costs occurring during the acute phase of the disease (i.e., hospital-related costs) and those associated with potential middle-to-long term sequelae. The hospital-related costs were derived, as stated above, from DRGs [38]. DRG fees are based on the length of hospital stay and include all inpatient costs for acute standard care and complications. Conversely, costs related to potential sequelae and to follow-up costs (for survivals both with and without sequelae) were taken from published studies [40, 46].

In the base case model we assumed a €67 vaccine cost per dose, corresponding to the price the vaccine is currently being sold in Italy [50, 51], and a vaccine administration cost per dose of €7, derived from an Italian study by Giorgi-Rossi et al [52]. We also included costs for adverse vaccine events, but not related QALY losses, since adverse reactions were assumed to be of short duration and without lifelong effects [44]. All costs were assessed at 2013 euro price level.

An infant immunisation strategy at 2, 3, 4 months with a booster dose between 12 and 23 months as indicated in Bexsero Summary of Product Characteristics [53] was considered. Immunisation coverage was assumed to be comparable to the current coverage for MenC vaccine in the two Italian regions (i.e., 80%).

Although 4CMenB showed to be immunogenic in infants [9] and adolescents [12], there is a lack of evidence on its real efficacy, since the vaccine had not yet been evaluated in efficacy trials or used routinely in any country worldwide. At the same time published data point out a little reduction in the 4CMenB immune response (or in those related to other vaccines) when the new 4CMenB is given in combination with other routine childhood vaccinations [54]. In the base case model we considered that a 75% vaccine efficacy was plausible according to experts’ opinion, published literature [2, 5], published clinical trials and experience derived from OMV vaccines [55, 56].

Vaccine trials, based on Serum Bactericidal Antibody (SBA), suggest a 100% strain coverage might be possible [9, 57], even though a recent phenotypic approach (MATS) indicates strain coverage could be lower (87% for Italy, CI 70–93%) [13, 58]. We decided to consider SBA activity the gold-standard assessment and to assume that the vaccine can protect against all meningococcal strains.

Published studies indicate that the protection provided by primary vaccine schedule in infants wanes rapidly [9, 53] and varies largely between the four vaccine antigens, thus hindering the assessment of the overall duration of protection. Data on the persistence of the antibody response following subsequent boosting are also limited [59]. We considered a base case duration of 1.5 years after 3 doses [5, 60] increasing to 3 years following the booster dose at 12 months on the basis of estimates from clinical trials [61, 62] and evidences from OMV vaccines [63].

Due to the lack of national recommendations indicating a reference ICER value to identify cost-effective public health interventions, a €40,000 per QALY gained threshold was used according to current NICE guidelines [64].

#### Sensitivity analysis

The most likely parameter values were used in the base case analysis. However, to test the robustness of the results obtained, both univariate and bivariate analyses were conducted looking at the effect of changing, respectively, one or two parameters at a time within a given range. In addition, a probabilistic sensitivity analysis (PSA) was performed using Monte Carlo simulation and drawing input parameter values from their probability distribution using Latin Hypercube sampling (see Tables 1 and 2 for details on distributions used and ranges). In particular, the following alternative scenarios were evaluated in the PSA: i) a base-case scenario, where all parameters values were sampled from their distributions except for discount rate, immunisation coverage, number of doses and cost per dose; ii) a scenario where discount rate was fixed at 1.5% for both costs and benefits in line with the NICE Public Health Guidelines 2013 [64]; iii) a third scenario where discount rate was assumed equal to zero (i.e., no-discounting); iv-ix) six low-cost-per-dose scenarios, where we hypothesized a €40 and €20 cost per dose at different discount rates (3% for both costs and benefits, 1.5% for both costs and benefits and no discounting). Moreover, we tested our analysis evaluating two high-incidence scenarios (x-xi), where incidence rates were assumed 3 and 6 times higher than the base case (i.e., as in other European countries [65]) (see S1 Fig for details of these additional scenarios).

Sensitivity and scenario analyses were performed using @RISK6 (Palisade Corporation, NY, US).

## Results

### Epidemiology of invasive meningococcal disease

**Fig 2:**
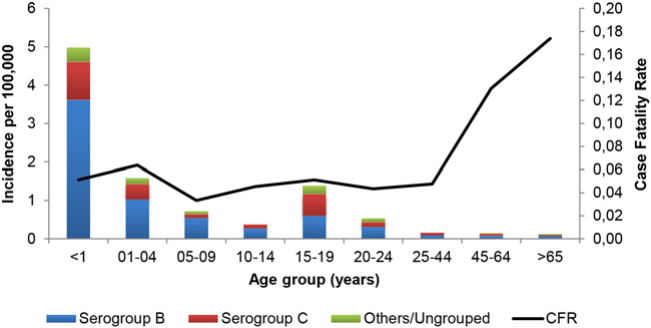
IMD incidence and case fatality rate. Average annual incidence and case fatality rate by age group for IMD in Lombardia and Piemonte over the six epidemiological years, 2007–2012.

A total of 341 IMD cases were reported during the 6-year study period (2007–2012), 244 of which occurred in Lombardia and 97 in Piemonte. 164 cases were male (48.1%) and 177 female (51.9%). The resulting overall disease incidence was 0.4/100,000/y, decreasing over the 6-year period and ranging between 0.28/100,000/y in 2012 to 0.58/100,000/y in 2009. Similarly, all the different serogroup-specific rates showed a decline after 2009 that was particularly evident for meningococcal serogroup C. The highest incidence was observed in infants <1 year (5.36/100,000/y) and in children aged 1–4 years (1.71/100,000/y), with a secondary lower peak in teenagers aged 15–19 years (1.42/100,000/y). Fig 2 shows IMD incidence by age classes according to capsular groups, revealing a comparable trend among the different serotypes and indicating that most cases occurred among young children.

Over the 6-year period, meningococcal capsular group B accounted for 59.24% of IMD cases (202/341 cases), followed by capsular group C with 29.62% (101/341). The aggregate number of the other capsular groups was 38 (Y: 18, A: 3, W: 11, other: 6) accounting for 11.14%. MenB-related disease incidence averaged at 0.21/100,000/y during the study period. Most of MenB cases occurred in infants <1 year and in young children 1–4 years, accounting for 3.61/100,000/y and 1.03/100,000/y, respectively. The average incidence then declined progressively until 15 years of age, where a second peak of disease was observed (0.60/100,000/year). In the older age classes incidence steadily decreased again reaching a minimum in people above 65 (0.09/100,000/y in 24–44 years, 0.08/100,000/y in 45–65 years, 0.07/100,000/y over 65 years).

All the IMD cases were hospitalised during the study period. The clinical manifestations of meningococcal infection were reported by the Hospital Discharge Form as meningitis (57%), sepsis (37%), both sepsis and meningitis (4%), and other symptoms or symptoms not specified (2%). Most of admissions occurred in infectious diseases units (47%), followed by paediatric units (31%), intensive care units (10%), neurological units (4%), and other units (8%).

A total of 22 in-hospital deaths were observed, accounting for an overall CFR of 0.074 and ranging from 0.057 in children ≤4 years to 0.17 in subjects ≥65 years (Fig 2).

### Cost-effectiveness analysis

#### Base case results

The model predicted 128 MenB disease cases and 8 deaths corresponding to: 76 MenB discounted cases per year over the lifetime of the 2012 birth cohort, an estimation of 36 cases avoided by introducing the routine early infant vaccination, and a total of 319 QALYs gained. Under our base case assumptions (i.e., 4 doses, an overall vaccine price of €296 including administration costs, 80% vaccination coverage, 100% strain coverage, 75% vaccine efficacy, 3 years vaccine protection and no herd immunity), the net costs of the programme would be almost €135 million (€34 million for Lombardia and Piemonte regions together).The resulting ICER of €376,042/QALY gained indicated that the immunisation programme is not deemed to be cost-effective, as its cost per QALY ratio is considerably above the threshold of €40,000.

#### Sensitivity analysis

**Fig 3:**
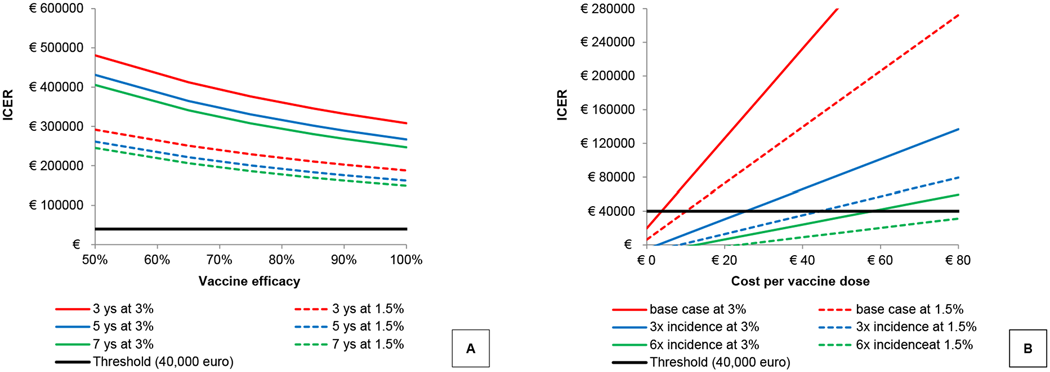
Bivariate analysis. Bivariate analysis: A: ICER values at varying vaccine efficacy (%) and duration of vaccine-induced immunity (years); B: ICER values at varying vaccine cost per dose (€) and base case incidence (multiplier).

The robustness of the above result was evaluated firstly performing a univariate sensitivity analysis and, subsequently, using a bivariate approach where two of the most critical identified model parameters were varied at a time. In Table 3, the univariate analysis results are presented according to the three alternative discount rates adopted (3%, 1.5% and no discounting). The bivariate analysis is presented in Fig 3, where ICER values are reported for different combinations of vaccine efficacy and duration of protection (range 3–7 years) on one side (Fig 3a), and vaccine cost per dose and a multiplier of base case incidence rates (up to 6x) on the other (Fig 3b). In both cases model results appear robust to parameters variation and the bivariate analysis indicated that the vaccine price per dose would need to be as low as €4 to obtain an ICER within the acceptable threshold of €40,000 per QALY gained. Only under the assumption of a lower discount rate (i.e., 1.5% for costs and benefits) or an IMD incidence rate 3 to 6 times higher than the base case value (i.e., similar to the one of some Northern European countries [65]) the ICER would end up being below the threshold. Considering the current MenB Italian incidence and 1.5% discount rate, the immunisation programme would be cost-effective only for a vaccine price below €10.

**Table 3:** ICER values (€) at the variation of each single base case parameter (min, max) under the three discount scenarios.

Parameters	Scenario 1	Scenario 2	Scenario 3
Base case	376,042	229,175	118,993
Disease incidence (lower bound) ^a^	699,548	436,788	236,704
Disease incidence (upper bound) ^a^	210,866	124,917	61,076
Case fatality rate (lower bound) ^a^	395,239	240,501	124,680
Case fatality rate (upper bound) ^a^	342,749	209,450	109,046
GP visit cost (lower bound) ^b^	376,042	229,175	118,993
GP visit cost (upper bound) ^b^	376,042	229,175	118,993
Costs acute stay, paediatric (lower bound) ^c^	376,475	229,455	119,153
Costs acute stay, paediatric (upper bound) ^c^	372,898	227,144	117,835
Costs acute stay, adult (lower bound) ^c^	376,103	229,234	119,047
Costs acute stay, adult (upper bound) ^c^	375,635	228,783	118,634
Follow-up cost for survivals (lower bound) ^b^ ^,^ ^d^	376,145	229,245	119,036
Follow-up cost for survivals (upper bound) ^b^ ^,^ ^d^	375,938	229,104	118,949
Cost for sequelae, annual (lower bound) ^b^	379,291	232,406	122,213
Cost for sequelae, annual (upper bound) ^b^	372,794	225,945	115,775
Proportion of any unilateral or bilateral hearing loss (lower bound) ^b^ ^,^ ^e^	380,387	231,833	120,379
Proportion of any unilateral or bilateral hearing loss (upper bound) ^b^ ^,^ ^e^	371,795	226,577	117,639
Proportion of mental retard (cognitive problem) (lower bound) ^b^ ^,^ ^e^	406,144	247,597	128,599
Proportion of mental retard (cognitive problem) (upper bound) ^b^ ^,^ ^e^	350,094	213,304	110,723
QALY loss for any unilateral or bilateral hearing loss (lower bound) ^b^ ^,^ ^e^	380,701	232,025	120,479
QALY loss for any unilateral or bilateral hearing loss (upper bound) ^b^ ^,^ ^e^	371,495	226,393	117,543
QALY loss for mental retard (cognitive problem) (lower bound) ^b^ ^,^ ^e^	405,438	247,166	128,373
QALY loss for mental retard (cognitive problem) (upper bound) ^b^ ^,^ ^e^	350,620	213,625	110,890
Vaccine efficacy (lower bound) ^b^	480,738	291,753	151,728
Vaccine efficacy (upper bound) ^b^	307,876	187,853	97,074
Strain coverage (lower bound) ^f^	411,137	250,270	130,090
Duration of protection after 4 doses (lower bound) ^b^	467,255	284,949	148,682
Duration of protection after 4 doses (upper bound) ^b^	343,704	208,995	108,050
Cost per vaccine dose (lower bound) ^b^	304,773	184,591	94,432
Cost per vaccine dose (upper bound) ^b^	447,311	273,759	143,554
Administration cost per dose (lower bound) ^b^	365,405	222,520	115,327
Administration cost per dose (upper bound) ^b^	386,679	235,829	122,659
Rate of mild adverse events (lower bound) ^b^	376,042	229,175	118,993
Rate of mild adverse events (upper bound) ^b^	376,042	229,175	118,993
Rate of anaphylactoid events (lower bound) ^b^	376,042	229,175	118,993
Rate of anaphylactoid events (upper bound) ^b^	376,042	229,175	118,993
Cost for those with mild adverse events (lower bound) ^b^	376,041	229,174	118,993
Cost for those with mild adverse events (upper bound) ^b^	376,042	229,175	118,993
Cost for those with anaphylactoid events (lower bound) ^b^	376,041	229,175	118,993
Cost for those with anaphylactoid events (upper bound) ^b^	376,042	229,175	118,993

ICER values (€) at the variation of each single base case parameter (min, max) under the three discount scenarios (Scenario 1: 3.% discount rate for both costs and benefits, Scenario 2: 1.5% discount rate for both costs and benefits, Scenario 3: no discounting).

^a^ Lower and upper bound of the disease incidence and the case fatality rate was calculated using the minimum and maximum value for each age class over the 6-year study period.

^b^ Lower and upper bound was equal to +/- 20% of the base case value.

^c^ Lower and upper bound of hospital costs was calculated using the minimum and maximum value acquired from the Italian Hospital Discharge Database.

^d^ Cost of follow-up for survivals both with and without sequelae was obtained using estimates from an Italian study by Lucioni [46].

^e^ Examples for some of the main sequelae.

^f^ Lower bound was drawn based on the estimate of the Italian strain coverage using the MATS assay (87%) [13].

#### Probabilistic sensitivity analysis

**Fig 4:**
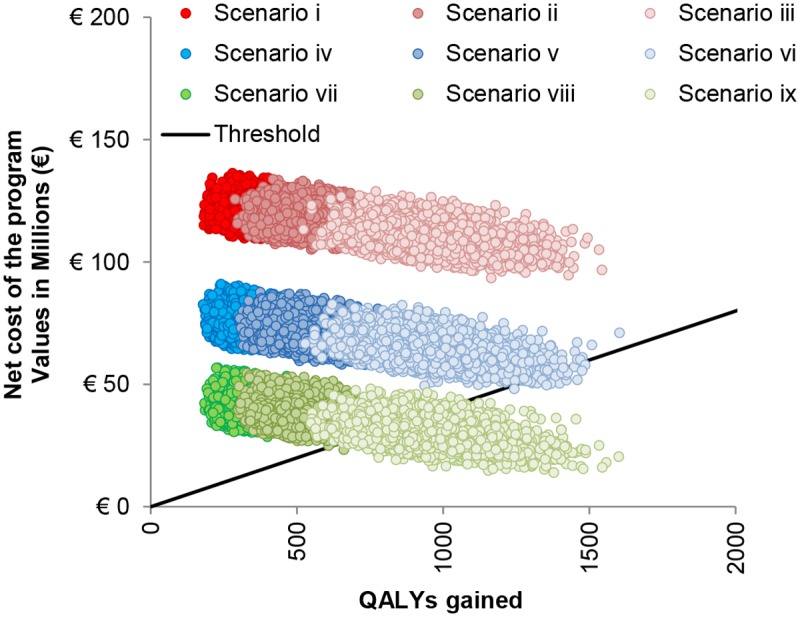
Multivariate analysis. Multivariate sensitivity analysis: results of the different scenarios. i) base-case scenario (all parameters values are sampled from their distributions except the discount rates, the immunisation coverage, the number of doses and the cost per dose; ii) discount-rate-1.5% scenario (discount rate is fixed at 1.5% for both costs and benefits); iii) no-discounting scenario (discount rate is assumed equal to zero); iv-ix) lower-cost-per-dose scenarios (iv: vaccine cost per dose €40 at 3% discount rates; v: vaccine cost per dose €40 at 1.5% discount rates; vi: vaccine cost per dose €40 at no discounting; vii: vaccine cost per dose €20 at 3% discount rates; viii: vaccine cost per dose €20 at 1.5% discount rates; ix: vaccine cost per dose €40 at no discounting).

Multivariate sensitivity analysis was conducted by sampling model parameters from pre-assigned probability distributions (5000 runs). Fig 4 shows the results of the multivariate sensitivity analysis carried out for the base case scenario (all parameters are varied except the cost per dose, the immunisation coverage, the number of doses and the discount rate) and for the main alternative investigated scenarios. As a result, only when considering a lower discount rate (i.e., either 1.5% or no discounting) together with a lower vaccine price per dose (i.e., €20) or a remarkable increase in the incidence rate of the MenB disease (S1 Fig) the model provided some cost-effective simulations (dots below the threshold line). In all the remaining scenarios none of the simulations yielded a cost-effective result (the combinations of net cost and QALYs gained were all above the threshold line indicating €40,000/QALY). Applying low discount rates, the multivariate sensitivity analysis showed that the programme could be cost effective for a vaccine price ≤€20.

## Discussion

Among the diseases preventable by immunisation, IMD remains a high public profile illness deserving the most rigorous consideration because of its rapid and severe onset, high mortality rate and burden of sequelae.

Similarly to other studies [1, 10], epidemiological data collected in our work indicate that infants in the first year of life experience the highest risk of infection, while two lower incidence peaks are observed in children between 1–4 years and among adolescents aged 15–19 years. Collected data retrace the epidemiology of meningococcal disease [1, 10] also for the mortality rate that shows an increase with age, resulting significantly higher among patients aged over 45 compared to those aged <45 years.

Our analysis shows Italy as a very low incidence country for meningococcal disease (0.4/100,000/y considering all meningococcal serogroups), so that the Italian IMD incidence results one of the most critical model parameters conditioning the cost-effectiveness of the novel vaccine. Even though meningococcal serogroup B is by far the main responsible of the disease, accounting for 59% of the cases, because of the low Italian incidence rate economic acceptability of the vaccine would be difficult to achieve. Moreover, as occurred in many high-income countries in the past decade [8, 27], also in our analysis the incidence of IMD showed a decreasing trend among all serogroups (from 0.58 to 0.28) over the last 6-year study period, even if fluctuations have been historically reported [10, 66] suggesting that future incidence remains still uncertain.

Cost-effectiveness results indicate that introducing MenB vaccine into the Italian routine infant immunisation schedule may prevent about one third of disease cases over the lifetime of a single vaccinated birth cohort. Nevertheless, assuming a 3% discount rate on both costs and benefits, it is unlikely that vaccination could be cost-effective (ICER <€40,000/QALY) at the current MenB Italian incidence, at any vaccine price per dose and even under different assumptions of the model parameters evaluated in the sensitivity analyses. Modification of the discount rates improves the cost-effectiveness of the immunisation programme leading to some cost-effective scenarios if the vaccine could reach competitive prices (i.e., ≤€20).

In the model we have used the epidemiological results of Lombardia and Piemonte to infer data for the whole Italian country. These are two of the most populated Italian regions, which cumulatively account for 14,217,000 inhabitants (i.e., a quarter of the entire Italian population). We checked the representativeness of the derived regional incidence and fatality rates, as well as vaccine-uptake, comparing these with the ones drawn from a study conducted at national level during the same period by the Italian National Institute of Health (ISS) [45]. We found that our data are perfectly consistent with national figures (see S1 File).

At present, several countries are considering the introduction of the novel 4CMenB vaccine for universal immunisation. Belgium, Ireland and Canada have not yet taken an official position [45], while in France, Germany, Spain and USA vaccination is expected to be introduced for high-risk groups only or in response to meningococcal serogroup B outbreaks [45]. In France [67], the Netherlands [2], Canada [68] and Spain [69], epidemiological data and economic models have shown that the introduction of the immunisation programme is unlikely to be cost-effective. Only the UK has recently recommended a programme for use of the MenB vaccine within the National Health System (NHS) infant immunisation schedule, as long as this can be achieved at a low price (i.e., <20% of the listed price) [44, 70]. This decision came after an interim statement by the Joint Committee on Vaccination and Immunisation (JCVI) [59], the UK Government independent committee on vaccine policy, which had originally advised against the introduction of the routine infant or adolescent immunisation since this was highly unlikely to be cost-effective at any vaccine price. However, with a final decision dated March 2014, which took account of stakeholders’ comments and more recent additional evidence, the JCVI assessed the vaccine to be cost-effective for infants when considering a series of favourable assumptions (i.e., potential litigation costs related to IMD, a quality of life adjustment factor, QALY losses in family and network members, QALY losses associated with the acute disease, and removal of the infant dose of meningococcal C vaccine).

Also, differently from our results, another Italian study [40], based on a model developed by Novartis, has recently evaluated the effectiveness and cost-effectiveness of Bexsero from both the societal and the NHS perspective. The study finds the programme to be deemed cost-effective under the assumptions of lower vaccine price per dose or lower discount rates pointing out more favourable results than ours. Unfortunately, though base line epidemiological and economic parameter values appear quite similar between the two studies, the results are difficult to compare due to the lack of information about vaccine efficacy and duration of protection. Moreover, even though QALY losses for long term IMD sequelae and their probabilities are taken from the same sources, the final value of incremental QALYs gained results significantly higher compared to our analysis as well as to other similar works [2, 5].

Our study presents some limitations which deserve to be discussed. First of all, our analysis was undertaken adopting only the NHS perspective. Though we are clearly aware that costs to society are relevant aspects that can strongly influence the overall outcome of an economic analysis, given the lack of robust Italian data we have decided to remain conservative and to evaluate only the direct cost to the public health payer.

Moreover, limited data are available and a large uncertainty exists around several key parameters, in particular those relating to the nature of the novel vaccine, including vaccine efficacy, duration of protection and strain coverage [71]. As undoubtedly each of these factors could affect the direct impact of the vaccine at individual level, they could also affect the overall effectiveness at population level. Nevertheless, we performed an extensive sensitivity analysis on our parameters and our results were confirmed under most assumptions.

The vaccine has the potential to provide broad coverage against most circulating MenB strains [12] although differences exist between countries [13, 58]. In particular, trials showed its efficacy is likely to depend on the number of antigens expressed by the different strains, increasing for strains that express more than one antigenic protein and decreasing for those carried only a single one [13]. National or even regional differences in strains represent a critical issue to predict the effectiveness of the intervention. Here we decided to assume a 100% strain coverage on the basis of evidence collected by using the SBA assay [9, 57], considering that this method has been accepted as the most appropriate measure to predict the effective coverage of vaccines against serogroup B meningococci [8]. Furthermore, recent data also suggests that MATS assay, the alternative available method to support implementation of meningococcal vaccines, underestimates the potential coverage of 4CMenB probably because of the inability to capture the synergetic effect of the different components of the novel vaccine [72]. Even though new evidence suggests that 4CMenB coverage might be lower than 100% (i.e., 88%) [72], we have decided to keep our assumption, since the new estimate is based only on the English and Welsh population while other papers [13] have highlighted that Italy probably experiences one of the highest strain coverage.

Differently from the recent position statement from the JCVI [70] and new published works [44], which agreed on a 95% short-term vaccine efficacy, we decided to set our base case efficacy level at 75%. Even though this could be considered a conservative assumption, we preferred to assume this value, largely referring to experts’ opinion, other related literature [2, 5], published clinical trials and assumptions derived from OMV vaccines [55, 56].

Nevertheless, the validity of our results was also confirmed when varying the distribution around the parameters reflecting their uncertainties.

We did not include herd immunity effects in our model. Although the novel vaccine and the past ones against meningococcal serogroup B have little, if any, impact on carriage [73, 74], this is one of the main determinants of indirect protection that the vaccine might provide. Ignoring herd immunity can clearly underestimate the positive externalities of the vaccination programme at the population level. Indeed, MenC vaccination has proved that herd protection is extremely important on vaccine effectiveness [75, 76]. There is evidence to suggest the novel vaccine can disrupt carriage, indicating that the impact of 4CMenB on the acquisition of nasopharyngeal meningococcal carriage is likely to be around 30% [74]. However, the magnitude of the real effect is difficult to be accurately predictable from these study findings [70, 71]. We believe that further information on whether 4CMenB can disrupt carriage and induce herd immunity will be necessary in the future to determinate the true potential effectiveness.

Similarly, serotype replacement was not considered in our analysis due to scarcity of data and on the basis of no evidence of replacement effects following introduction of MenC vaccination in Italy although there are some concerns about this in other countries. Development and progress of replacement could obviously have a negative impact on vaccine effectiveness.

All costs and health benefits were discounted at a 3% annual rate in the base case analysis according to the Italian guidelines [42, 43] though sensitivity analysis was performed looking at different scenarios on discounting. Undoubtedly discount rate has a great impact in the models on ICER. There are discordant opinions on how and whether to use discounting health benefits in economic evaluations [77] and in particular some authors believe that future benefits of preventive health programmes should not be discounted [78, 79]. We can argue that also NICE guidelines [64] appeared not so clear about the topic, suggesting a discount rate of 3.5% for both costs and benefits in case of health technology assessments, and 1.5% for both in case of public health interventions.

Our analysis showed the price of the vaccine as one of the most critical factors which could influence the cost-effectiveness of the programme. We decided to assume a vaccine price of €67 in the base case analysis. Indeed, this is the price at which the vaccine is currently being sold to those Italian local health authorities that have independently decided to offer it [50, 51]. Unfortunately, the current situation in Italy for vaccine introduction is very heterogeneous and each local health authority has the possibility to purchase individually the product. This may affect both the epidemiological outcome of the programme and the costs at which the vaccine is sold. If more coordination between regions was introduced, we anticipate possible reductions of the vaccine price and, as a consequence, lower ICER values.

Even though 4CMenB vaccine was designed to protect against meningococcal serogroup B, the protein antigens of the vaccine are also present in non-group B strains, thus it is expected that the vaccine could offer some protection also against the other serogroups. However, we decided to consider no cross-protection as there is still too limited evidence.

Recently it has been suggested that no difference on antibody titres against the recombinant protein would be observed after two doses 2 months apart, as opposed to three doses one month apart, indicating that two doses during the first year of life (2 and 4 months) with a booster dose at 12–23 months should likely provide substantial protection against MenB in infants [70]. This could not only positively affect the cost-effectiveness results but also the parental acceptance, which represents one of the most critical factors to consider before the introduction of a new vaccine into the NIP. Different studies have shown that the number of immunisations and shots given may condition the rate of deferral doses, thus reducing the immunisation coverage [80] (there is clearly variability between individuals, but it is reasonable to say that both parents and clinicians usually agree only to a finite number of infant vaccine injections at a single visit).

In the same pathway, clinical trials [53] have suggested that, although immune responses to routine vaccines were much the same with or without 4CMenB, concomitant vaccination was associated with increased reactogenicity [9]. In particular fever and febrile seizures would be more frequent and pronounced if 4CMenB was given with other routine immunisations. Even though studies demonstrated that this adverse effect could be prevented by prophylactic administration of antipyretics [81], the potential impact on parental acceptance of the routine immunisation programme must be considered.

Due to the lack of national recommendations indicating an ICER reference value below which public health interventions can be considered cost-effective, we adopted the current NICE guidelines [64] and used a €40,000/QALY gained threshold. The NICE guidelines use a £20,000–30,000/QALY upper limit of cost-effectiveness range and several moderators can affect the final decision, meaning that this is not static or invariable. The JCVI actually decides to make recommendation in relation to vaccines using a limit of £20,000/QALY. In most European countries there is no formal threshold and €20,000–30,000 per QALY is often mentioned for new vaccine programmes to indicate highly favourable cost-effectiveness results [82]. On the contrary, programmes are generally not considered to be cost-effective for ICER values over €50,000 per QALY gained [83, 84].

It should be also considered that the newly licensed MenB vaccine seems to be the first immunisation programme to be evaluated through an HTA process before being introduced into the Italian routine infant immunisation schedule. This clearly represents a step further in the framework of policy decision making, but it also highlights the notably different criteria adopted with respect to previous vaccination policies. Therefore it appears extremely difficult to establish a cost-effectiveness threshold for this vaccine without checking if and how other programmes might have respected the same condition.

The experience related to the post-market evaluation of the vaccine against meningococcus C has proved the importance of having a good post-vaccination surveillance in order to monitor the real clinical effectiveness of the drug [8]. Good active population-based sentinel surveillance would allow to gather important information also about the 4CMenB capacity to disrupt carriage and reach the herd immunity. Without any doubt more detailed information about the effective vaccine efficacy, duration of protection, reactogenicity, posology and serotype replacement, as well as the level of vaccine coverage are essential to be achieved.

## Conclusions

Our study has highlighted different critical issues related to the potential introduction of the new meningococcal serogroup B vaccine (Bexsero) into the Italian health market arena.

The introduction of this new vaccine in Italy deserves considerable attention because the disease involves young infants exerting a strong public interest. Also, this vaccine would be the first one to be introduced into the Italian routine infant immunisation schedule on the basis of a formal HTA evaluation process at national level. Up to now, Italy has proved to be a very heterogeneous country in the field of vaccinations as a result of the regional health system decentralisation.

Italy is certainly an extremely low incidence country for meningococcal disease. With such an incidence it is very unlikely that the vaccine would be deemed cost-effective, especially at the price that is currently proposed for the release on the Italian market. However, it is important to underline that our model estimates the new MenB vaccine might be able to prevent about one third of the cases of meningitis in the general population. This figure could be even more important in case the vaccine proved its efficacy against carriage acquisition. In this sense further evaluation in the adolescent age group would be important to acquire.

4CMenB, as it seems, will be administered during the first year of life through three doses with a possible later booster dose [53], which means adding at least three more doses in the first twelve months of life. How to implement this modified routine immunisation schedule is not a secondary problem for the load of organisational system, the financial resources that would be needed to carry out the programme and the parental adherence (with a possible decreasing compliance also to the already existing vaccination schedule).

Even though numerous uncertainties remain about some vaccine parameters (i.e., efficacy, duration, safety and possibility of causing replacement), conditioning the complexity associated with modelling the impact of the new vaccine, clinical trials have demonstrated significant benefits at the population level. At the same time the new 4CMenB vaccine might have a positive impact if offered selectively to certain high-risk groups of the population or used during meningococcal serogroup outbreaks.

Surely a close collaboration between public health institutes and manufacturers in all these fields is to be desirable as well as a continuous intensive surveillance of meningococcal diseases pre and post vaccine introduction.

## Supporting Information

S1 Fig PSA results for high-incidence scenarios. Multivariate sensitivity analysis: results for the high-incidence scenarios x and xi (incidence rates were assumed 3 and 6 times higher than the base case).
(TIF)

S1 Dataset Dataset. (PDF)
