# Supplementary figures and images for: Health and Economic Outcomes of Introducing the New MenB Vaccine (Bexsero) into the Italian Routine Infant Immunisation Programme
Source: PLoS One. 2015 Apr 13;10(4):e0123383. doi: 10.1371/journal.pone.0123383 (PMC4395261; doi:10.1371/journal.pone.0123383)

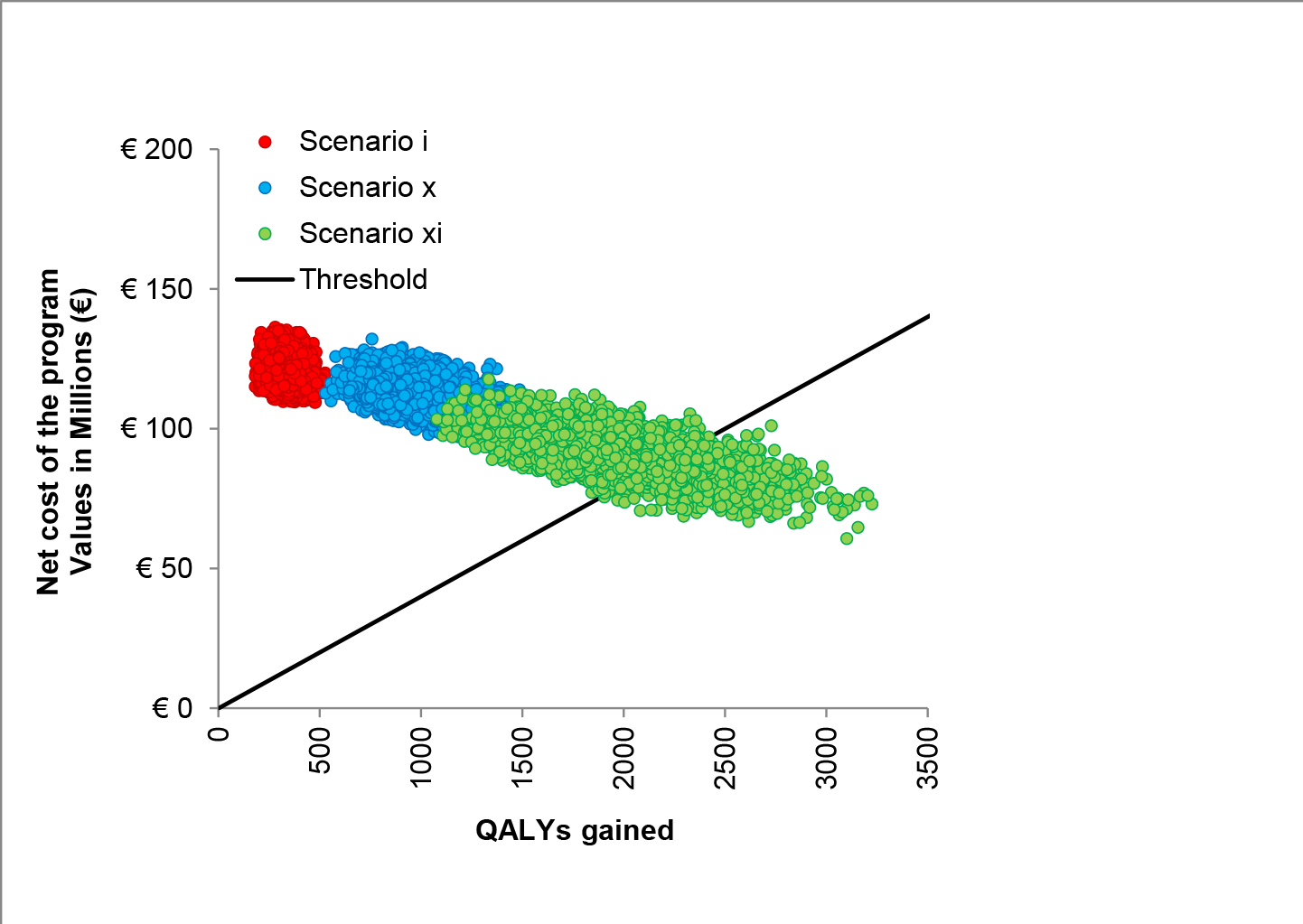

Supplement: S1 Fig — Multivariate sensitivity analysis: results for the high-incidence scenarios x and xi (incidence rates were assumed 3 and 6 times higher than the base case). (TIF) [file pone.0123383.s001.tif]
