# Supplementary material for: Health and Economic Outcomes of Introducing the New MenB Vaccine (Bexsero) into the Italian Routine Infant Immunisation Programme
Source: PLoS One. 2015 Apr 13;10(4):e0123383. doi: 10.1371/journal.pone.0123383 (PMC4395261; doi:10.1371/journal.pone.0123383)
Supplement: S1 Dataset — (PDF) [file pone.0123383.s002.pdf]

Population figures for Lombardia and Piemonte by year			
**Year**	**Lombardia**	**Piemonte**	**Lombardia & Piemonte**
2007	9,545,441	4,352,828	13,898,269
2008	9,642,406	4,401,266	14,043,672
2009	9,742,676	4,432,571	14,175,247
2010	9,826,141	4,446,230	14,272,371
2011	9,917,714	4,457,335	14,375,049
2012	9,700,881	4,357,663	14,058,544

Population figures for Lombardia and Piemonte by age group over the 6-year study period			
**Age group**	**Lombardia**	**Piemonte**	**Lombardia & Piemonte**
<1	574,165	228,725	802,890
1-4	2,292,826	920,767	3,213,593
5-9	2,737,652	1,123,869	3,861,521
10-14	2,598,291	1,096,968	3,695,259
15-19	2,566,240	1,106,534	3,672,774
20-24	2,703,382	1,180,208	3,883,590
25-44	17,525,082	7,453,783	24,978,865
45-64	15,659,046	7,287,423	22,946,469
65+	11,718,575	6,049,616	17,768,191

Average annual length of hospital stay (days) by age group for IMD in Lombardia and Piemonte over the 6-year study period	
**Age group**	**Lombardia & Piemonte**
<1	10.92
1-4	12.29
5-9	10.88
10-14	10.15
15-19	12.76
20-24	13.90
25-44	28.34
45-64	22.08
65+	22.25

Average annual cost of acute stay (€) for IMD in Lombardia and Piemonte over the 6-year study period			
	**Mean**	**Min**	**Max**
**paediatric (≤18 years)**	6,800	2,500	38,000
**adult (>18 years)**	8,250	2,700	45,000

Overall IMD incidence (per 100,000) by year in Lombardia and Piemonte	
**Year**	**Lombardia & Piemonte**
2007	0.42
2008	0.51
2009	0.58
2010	0.29
2011	0.35
2012	0.28

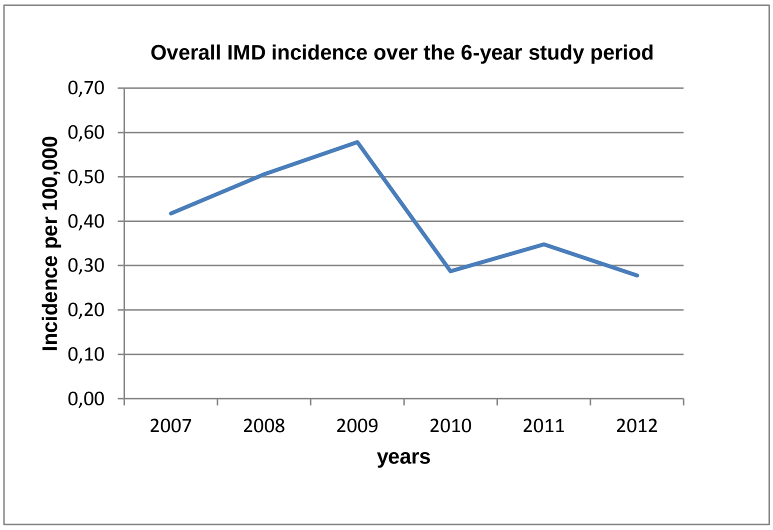

IMD incidence (per 100,000) by serogroup and year in Lombardia and Piemonte			
Year	MenB	MenC	Others/Ungrouped
2007	0.19	0.12	0.09
2008	0.23	0.20	0.05
2009	0.32	0.17	0.02
2010	0.19	0.04	0.04
2011	0.19	0.06	0.03
2012	0.15	0.06	0.01

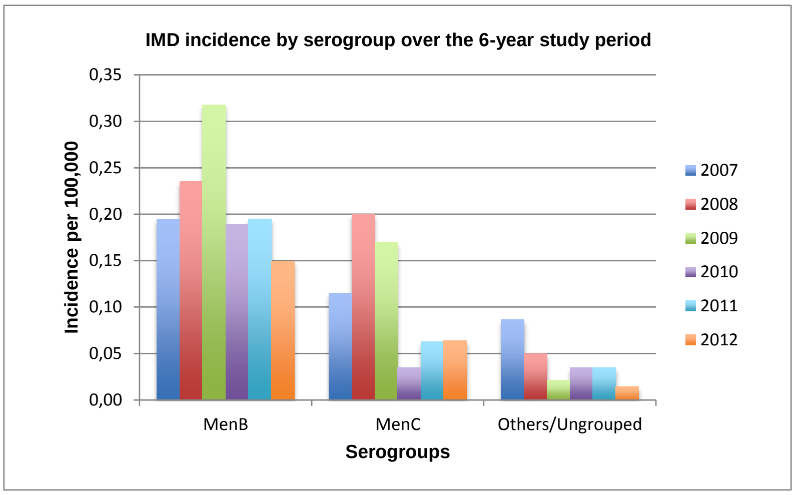

Average annual incidence (per 100,000) by serogroup and age group in Lombardia and Piemonte over the 6-year study period			
Age group	MenB	MenC	Others/Ungrouped
<1	3.61	1.00	0.37
1-4	1.03	0.40	0.16
5-9	0.54	0.10	0.08
10-14	0.27	0.11	0.00
15-19	0.60	0.57	0.22
20-24	0.31	0.13	0.10
25-44	0.09	0.07	0.00
45-64	0.08	0.06	0.02
65+	0.07	0.03	0.03

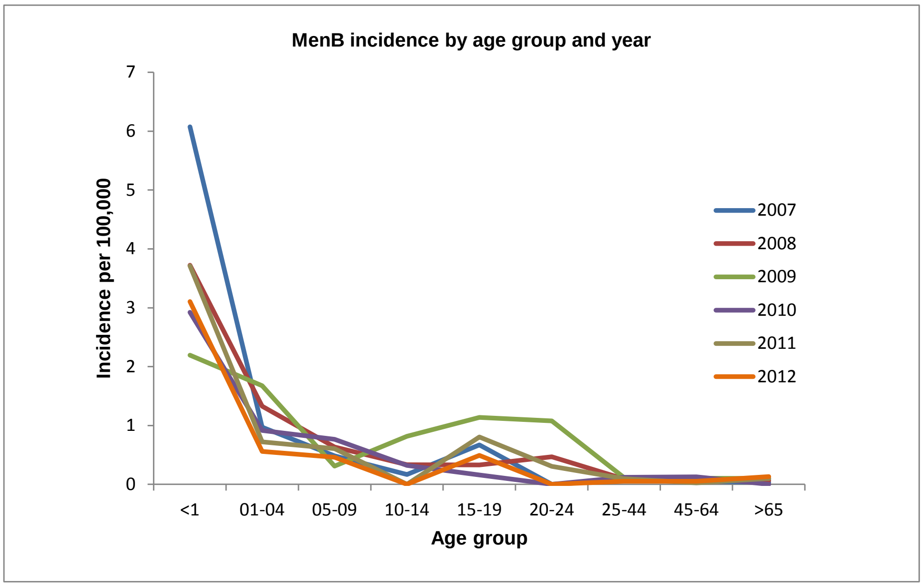

Average annual Case Fatality Rate (CFR) by age group for IMD in Lombardia and Piemonte over the 6-year study period	
**Age group**	**Lombardia & Piemonte**
<1	0.05
1-4	0.06
5-9	0.03
10-14	0.05
15-19	0.05
20-24	0.04
25-44	0.05
45-64	0.13
65+	0.17

Comparison between data from our study (Lombardia and Piemonte) vs data from the Italian National Institute of Health (whole Italian country)*		
**MenB annual incidence (per 100,000) by age group over the 6-year study period**		
**Age group**	**Lombardia & Piemonte**	**Italy**
<1	3.61	3.44
1-4	1.03	1.07
5-9	0.54	0.50
10-14	0.27	0.27
15-19	0.60	0.57
20-24	0.31	0.32
25-49	0.09	0.07
50+	0.09	0.11
**Overall MenB incidence and Case Fatality Rate over the 6-year study period**		
	**Lombardia & Piemonte**	**Italy**
**Incidence**	0.21	0.23
**CFR**	0.07	0.07

* Istituto Superiore di Sanità - Gruppo di Lavoro del CNESPS (2014). Dati e evidenze disponibili per l'introduzione della vaccinazione anti-meningococco B nei nuovi nati e negli adolescenti. Available: http://www.epicentro.iss.it/temi/vaccinazioni/pdf/Istruttoria%20MENINGOCOCCO%20B.pdf. Accessed 01 November 2014.
